# Successful Surgical Treatment of a Brain Stem Hydatid Cyst in a Child

**DOI:** 10.1155/2020/5645812

**Published:** 2020-01-23

**Authors:** Rahaf Alok, Jaber Mahmoud

**Affiliations:** ^1^Faculty of Medicine, Damascus University, Damascus, Syria; ^2^Department of Pediatrics, Faculty of Medicine, Damascus University, Damascus, Syria

## Abstract

Hydatid disease is a parasitic infestation, which is endemic in the Mediterranean region. It is often located in the liver and the lungs, whereas brain stem hydatid cysts are extremely rare. We report a case of a five-year-old female who presented with hemiparesis, and after investigations, she was diagnosed with a hydatid cyst in the pons. She also had cysts in her liver and kidney. The cerebral cyst was completely removed without rupture, using gentle water-jet dissection (Dowling's technique). She was feeling well after 4-month follow-up. We emphasize the importance of keeping hydatid cysts in the differential diagnosis of pediatric infratentorial cystic lesions.

## 1. Introduction

Hydatidosis is a zoonotic disease caused by the tapeworm of the complex Echinococcus sensu lato that is classified into 10 genotypes (G1-G10) [1]. People become infected by ingesting parasite's ova in contaminated fresh vegetables, domestic water, or after direct contact with dogs [2–4]. The Middle East is one of the highly prevalent region, but until now, there is no epidemiologic studies about the prevalence in Syria [2].

Hydatid cysts (HC) could be found in any organ of the body. While the liver and lungs are the most affected, brain stem infection is considerably rare [5]. We report a case of a hydatid cyst in the pons in a 5-year-old female that was treated surgically without rupture.

## 2. Case Presentation

A previously healthy 5-year-old female, living in a rural area in Syria but does not have direct contact with animals, presented with right hemiparesis that had started 2 weeks earlier. She had also complained of headache, fever, and epigastric pain during the last month, but no vomiting was experienced.

She was conscious but asthenic. The physical exam revealed right eye esotropia and ptosis, symmetrical dilated but reactive pupils, right-sided deviation of her mouth's angle, hyperreflexia and weakness in the right side, positive Babinski reflex in her right foot, and mild ataxia.

**Figure 1:**
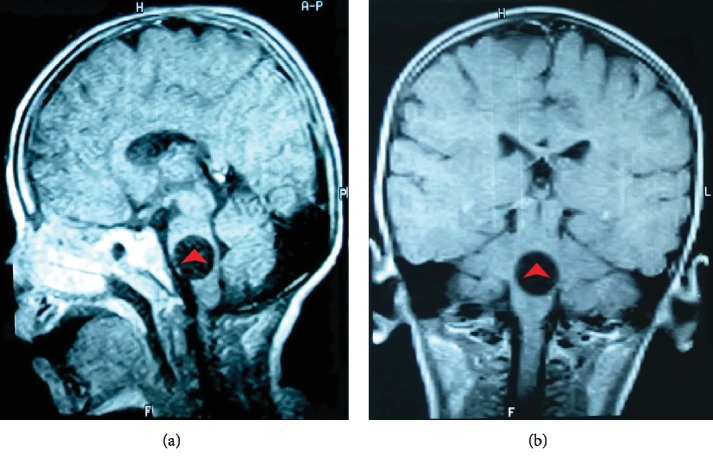
Noncontrast T1-weighted MRI shows round, well-circumscribed, isointense cyst (red arrowheads). (a) Sagittal image. (b) Coronal image.

**Figure 2:**
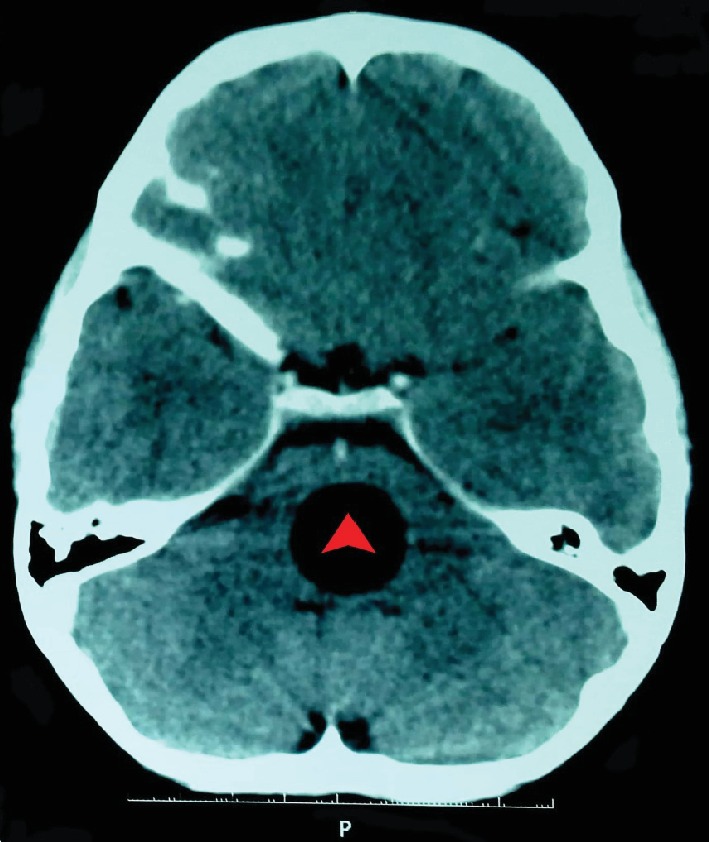
Enhanced CT scan shows a well-defined, thin-walled, round cyst in the pons (red arrowhead). No calcification, surrounding edema, or contrast enhancement is seen.

Brain magnetic resonance imaging (MRI) showed a well-defined round lesion in the pons. It was 21 mm in measurement, isointense to CSF, and did not enhance the contrast. There was no edema or displacement of the midline structures (Figure 1). Brain computed tomography (CT) confirmed the presence of sharply defined cystic lesion in the pons (Figure 2). Pan-CT scan revealed many small round cysts with smoothly demarcated walls in the liver, along with a small cyst in the left kidney.

The Echinococcus indirect hemagglutination test was positive. Liver enzymes, creatinine, and urea were all normal. The diagnosis of multiple organ involvement with hydatid cysts was highly suspected, and surgical intervention was planned.

Suboccipital craniotomy while in the prone position was performed. The dura was opened in a Y-shaped manner. The vermis was split in order to reach the 4th ventricle. The floor of that ventricle was gently dissected along the midline to reach the cyst. Dowling's technique was used to dissect the cyst, which included instillation of normal saline between the hydatid cyst and the surrounding tissue. Using this gentle water-jet dissection, surgeons were able to remove the cyst unruptured. After that, the pathology report showed a hydatid cyst wall without protoscoleces.

Albendazole was started 2 days before surgery at a dose of 7.5 mg/kg twice daily and was continued for 4 courses of 4 weeks separated by intervals of 2 weeks.

After surgery, the patient's strength of the upper limb improved, but she developed mild lower extremity weakness that improved over the follow-up period of four months.

Regarding the cysts that are located in other organs, we advised the patient to repeat the abdominal and chest CT after finishing her chemotherapy, but she did not.

## 3. Discussion

Cerebral hydatid cysts are a rare presentation of an endemic disease. They represent 1-4% of all hydatid cysts [6]. They are most commonly found in the middle cerebral artery territory, especially the parietal lobes [5], whereas brain stem infection is reported in only few cases [5].

They are usually solitary and primary [5]. Childhood is the most prevalent age group at which cerebral hydatid cysts are seen [5]. This case highlights the importance of keeping hydatid disease in mind when facing pediatric infratentorial cysts.

Radiology plays a key role in the diagnosis of hydatid cysts, although histopathological examination remains the gold standard [7]. Their typical imaging appearance is a single, large, spherical, thin-walled cyst without edema, or enhancement. On MRI, cyst fluid is isointense with CSF on T1WI and T2WI [8].

The cyst and the pericyst are characteristic imaging components of the HC, where the pericyst is the capsule of the cyst and it is best observed on MRI [8].

The differential diagnosis should include pilocytic astrocytoma that enhances on both CT and MRI and has a distinctive feature which is the existence of a contrast enhancing nodule [8]. Arachnoid cysts are extra-axial in location, and they are not as round regarding that there is no surrounding brain tissue [8]. Abscesses have a thick wall, which vividly enhances with contrast, and they are surrounded by edema [8, 9]. In neurocysticercosis, multiple cysts are usually seen in different stages, and indeed, they have a pattern called “dot in a hole” since the larval scolex could be present as a mural nodule [8].

Serological tests are usually negative in primary cerebral hydatid cysts; however, they become positive in case of associated visceral lesions. Regarding that those tests returns negative after surgical resection, they could be useful as an evidence of recurrence [10, 11].

Surgery is the mainstay of intracranial hydatid cyst treatment, and surgeons should do their best to remove them in toto without spillage [5]. Dowling's technique is the most accepted method nowadays. It requires a great osteoplastic flap, cautious handing during all the surgical steps, wide incision over the cyst, sloping the head of the table downward, and injecting saline between the cyst and the brain [12]. For vital areas like the brain stem, many surgeons prefer to aspirate the cyst then they remove its membrane; however, in our case, Dowling' technique was effective in the brain stem [13]. In a retrospective study, intraoperative rupture was reported in 26.3% of cases [7]. In order to minimize the risk for recurrence after rupture, surgeons should generously wash the surgical site with hypertonic saline and apply chemotherapy such as Albendazole or Mebendazole [5]. Postoperative mortality rate was estimated as 10%, and it was significantly higher in patients whose cysts ruptured or punctured during surgery [5].

Treatment with medications is required in patients experiencing recurrence, rupture during surgery, or have systemic hydatid disease. It could also be used preoperatively to reduce the size and number of multiple cysts, and postoperatively as a prophylactic treatment [5, 7]. Medication alone could be applied to treat inoperable cases usually as a palliative treatment; however, some cases were successfully cured using Albendazole alone [14].

## 4. Conclusions

Hydatid cyst should be kept in mind as a differential diagnosis of brain stem cystic lesions in endemic areas, especially in pediatric population.
